# Anti-Inflammatory and Anti-(Lymph)angiogenic Properties of an ABCB5+ Limbal Mesenchymal Stem Cell Population

**DOI:** 10.3390/ijms25179702

**Published:** 2024-09-07

**Authors:** Berbang Meshko, Thomas L. A. Volatier, Johanna Mann, Mark A. Kluth, Christoph Ganss, Markus H. Frank, Natasha Y. Frank, Bruce R. Ksander, Claus Cursiefen, Maria Notara

**Affiliations:** 1 Department of Ophthalmology, Faculty of Medicine, University Hospital Cologne, University of Cologne, 50937 Cologne, Germanythomas.volatier@uk-koeln.de (T.L.A.V.); johanna.mann@uk-koeln.de (J.M.); claus.cursiefen@uk-koeln.de (C.C.); 2 RHEACELL GmbH & Co. KG, Im Neuenheimer Feld 517, 69120 Heidelberg, Germany; andreas.kluth@rheacell.com (M.A.K.); christoph.ganss@rheacell.com (C.G.); 3 Transplant Research Program, Boston Children’s Hospital, Boston, MA 02115, USA; markus.frank@childrens.harvard.edu; 4 Harvard Skin Disease Research Center, Department of Dermatology, Brigham and Women’s Hospital, Boston, MA 02115, USA; 5 Harvard Stem Cell Institute, Harvard University, Cambridge, MA 02138, USA; nyfrank@bwh.harvard.edu; 6 School of Medical and Health Sciences, Edith Cowan University, Perth, WA 6027, Australia; 7 Department of Medicine, VA Boston Healthcare System, Boston, MA 02132, USA; 8 Division of Genetics, Brigham and Women’s Hospital, Boston, MA 02115, USA; 9 Massachusetts Eye & Ear Infirmary, Schepens Eye Research Institute, Boston, MA 02114, USA; bruce_ksander@meei.harvard.edu; 10 Center for Molecular Medicine Cologne (CMMC), University of Cologne, 50937 Cologne, Germany; 11 Cluster of Excellence Cellular Stress Responses in Aging-Associated Diseases (CECAD) Research Center, Joseph-Stelzmann-Str. 26, 50931 Cologne, Germany

**Keywords:** cornea, mesenchymal stem cells, ABCB5, (lymph)angiogenesis

## Abstract

Corneal transparency and avascularity are essential for vision. The avascular cornea transitions into the vascularized conjunctiva at the limbus. Here, we explore a limbal stromal cell sub-population that expresses ABCB5 and has mesenchymal stem cell characteristics. Human primary corneal stromal cells were enriched for ABCB5 by using FACS sorting. ABCB5+ cells expressed the MSC markers CD90, CD73, and CD105. ABCB5+ but not ABCB5− cells from the same donor displayed evidence of pluripotency with a significantly higher colony-forming efficiency and the ability of trilineage differentiation (osteogenic, adipogenic, and chondrogenic). The ABCB5+ cell secretome demonstrated lower levels of the pro-inflammatory protein MIF (macrophage migration inhibitory factor) as well as of the pro-(lymph)angiogenic growth factors VEGFA and VEGFC, which correlated with reduced proliferation of Jurkat cells co-cultured with ABCB5+ cells and decreased proliferation of blood and lymphatic endothelial cells cultured in ABCB5+ cell-conditioned media. These data support the hypothesis that ABCB5+ limbal stromal cells are a putative MSC population with potential anti-inflammatory and anti-(lymph)angiogenic effects. The therapeutic modulation of ABCB5+ limbal stromal cells may prevent cornea neovascularization and inflammation and, if transplanted to other sites in the body, provide similar protective properties to other tissues.

## 1. Introduction

Corneal transparency is crucial for good vision. The cornea consists of five layers (from anterior to posterior), i.e., the epithelium, Bowman’s layer, stroma, Descemet’s membrane, and endothelium [1]. A malfunction or damage of any of these layers may lead to a corneal disorder and loss of corneal transparency. The stroma comprises 90% of the thickness of the cornea. Due to its biomechanical structure, mostly a precise organization of collagen fibrils (Collagen I and V) arranged in lamellae, it provides transparency and mechanical strength [2,3,4]. Collagen fibrils are synthesized by keratocytes, the main cell type of the stroma, mostly residing in the anterior stroma [5].

**Figure 1:**
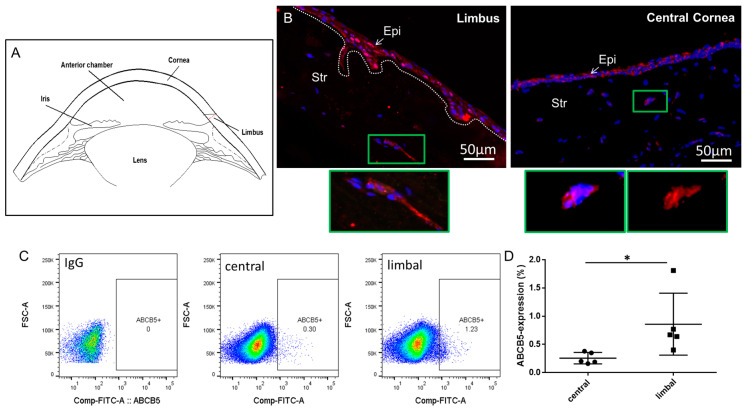
ABCB5 is expressed in limbal and central corneal stromal cells. (**A**) A schematic of the cornea cross section depicting the locations of the limbus, cornea, lens, and iris. (**B**) Immunocytochemistry of limbal and central corneal sections, red: ABCB5 (alexa 555) blue: DAPI. In green frame: magnified insets depicting clusters of ABCB5-expressing stromal cells in the limbal stroma and cornea stroma, respectively. Images captured using 20x magnification. (**C**) ABCB5 expression evaluated by FACS analysis of expanded stromal cells from the central cornea and the limbus. (**D**) Quantification of FACS data comparing the percentage of ABCB5-expresing cells derived from the central cornea and the limbus (* signifies *p* < 0.05, n = 5).

Stem cell (SC) populations within both the epithelial and the limbal stromal compartment were previously reported. A population of limbal epithelial stem cells (LESC) is located in the basal epithelial layer of the limbus, the physical junction of the avascular cornea, and the heavily vascularized conjunctiva (the location is depicted in a schematic of the cornea cross section, Figure 1A). Upon injury or due to normal turnover of the corneal epithelium, LESC enter the transient amplifying (TA) state while they migrate to the central cornea [1,6,7,8,9,10]. ATP-binding cassette, sub-family B, member 5 (ABCB5) [11] is considered a highly specific LESC marker [12]. In addition, we have recently reported a novel dual, context-dependent role of ABCB5 in the corneal inflammatory (lymph)angiogenesis response in a murine model and also by in vitro assessment of ex vivo cultured human limbal epithelial cells. Specifically, ABCB5+ LESCs were shown to inhibit inflammatory (lymph)angiogenesis during development of the cornea but promoted this same process in adult mice while also exerting anti-inflammatory effects. These findings are of high clinical relevance to LESC therapy against blindness [13].

The cornea is also home to another adult stem cell population, residing in the corneal stroma [14,15,16]. Corneal stromal stem cells, which are essentially putative limbal mesenchymal stem cells (MSC), are mainly distributed in the anterior part of the corneal stroma near the LESCs [17,18]. These MSC display the ability to self-renew, an important property of multipotent stem cells [19,20]. Previous reports confirm that these MSCs can be differentiated into functional keratocytes in vitro, which is critical for maintaining corneal transparency and producing a healthy corneal stroma [21,22,23]. These MSCs are therefore an important component of the limbal microenvironment and should be considered for use in therapies of ocular surface reconstruction and corneal tissue engineering [24].

Under normal conditions, the cornea is void of immune cell infiltrates and vessels. However, inflammatory challenge (disease or injury) leads to persistent inflammation and scarring. Like observed in other tissues, including the lung and the heart, which respond strongly to inflammatory stimuli, these adverse conditions reduce the cornea regenerative capacity and its ability to restore avascularity and transparency [25]. For this reason, the use of MSC has been proposed as a therapeutic strategy against corneal scarring and to promote cornea transplantation survival [26,27].

So far, dermal ABCB5+ MSC have been isolated and characterized with the goal of applying their key immunoregulatory properties to promising clinical applications [28]. ABCB5+ MSC are currently used in two clinical trials, testing their therapeutic potential against epidermolysis bullosa and refractory chronic venous ulcers [29,30,31,32].

In this study, we show first evidence of an ABCB5+ putative stem cell population within the corneal stroma. First, we established that these cells exhibited MSC characteristics by confirming their ability for trilineage differentiation, MSC marker expression, and superior colony-forming unit efficiency compared to their negative counterparts. Subsequently, we aimed to understand their potential immunoregulatory and angiogenic effects by carrying out analysis of secreted proteins. Functional assays were also carried out utilizing both blood and lymphatic endothelial cells cultured in either ABCB5+ stromal cell supernatants or their negative counterparts. Lastly, immunoregulation was functionally assayed by measuring the ability of ABCB5+ cells to hamper T-cell proliferation in a co-culture with Jurkat cells. Our results demonstrate that this population may play a key role in the immune- and (lymph) angio-modulation of the limbal transition zone between avascular cornea and heavily vascularized conjunctiva. Moreover, these stem cells may also be a potential therapeutic target to control pathogenic inflammation of (lymph) angiogenesis.

## 2. Results

### 2.1. ABCB5 Is Expressed in the Human Limbal/Corneal Stroma

The expression of ABCB5 in the human corneal stroma was assessed by immunocytochemistry on sections. Figure 1B depicts immunostaining of ABCB5 in representative human limbal and central corneal sections. In both limbus and central cornea, clusters of stromal cells express ABCB5 (Figure 1B, inlets depict respective magnified images of the marked green rectangles). The expression of ABCB5 in ex vivo cultured central cornea and limbal stromal cells was also confirmed by FACS analysis representative data (depicted in Figure 1C). In these ex vivo expanded cultures, the percentage of ABCB5-expressing cells derived from the central cornea (0.25 ± 0.1%) was significantly lower (*p* < 0.05) compared to those extracted from the limbus (0.86 ± 0.4%).

### 2.2. FACS-Sorted ABCB5+ Limbal Fibroblastic Cells Exhibit MSC Characteristics

**Figure 2:**
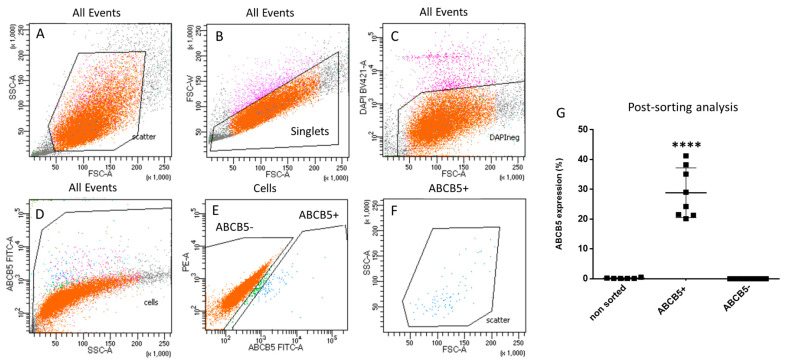
FACS sorting gating strategy for selection of ABCB5+ cells. (**A**) Cells separation from debris (forward scatter-FSC/side scatter-SSC dot-plot). (**B**) Exclusion of doublets. (**C**) Selection of live cells and (**D**,**E**) definition of ABCB5+ and ABCB5- cells. (**F**) Back-gating of the ABCB5+ gate to the SSC and FSC plot. (**G**) Post-sorting FACS analysis results (**** signifies *p* < 0.0001, n = 8).

FACS sorting was used to separate ABCB5+ and ABCB5- ex vivo cultured stromal cells. Importantly, the cells had to be expanded for one passage to obtain a sufficient number of cells for sorting. The sorting strategy is illustrated in Figure 2A–F: first, cells are separated from debris in the forward-(FSC-A)/side-scatter (SSC-A) dot-plot (Figure 2A) and subsequently, the doublets are also excluded (Figure 2B). The live cells (DAPI-negative, Figure 2C) are then selected in an additional gate for cells (Figure 2D) prior to finally defining two ABCB5+ and ABCB5- gates (Figure 2E). Figure 2F depicts back-gating of the ABCB5+ gate to the SSC and FSC plot. Post-sorting analysis FACS data demonstrated an average enrichment of 28.8 ± 8.35% in the ABCB5+ fraction vs. 0.18 ± 0.16% in the unsorted and 0% in the ABCB5- fraction (Figure 2G).

Subsequent to sorting, the ABCB5+ and ABCB5- fractions were plated separately, fed with SCM media, and expanded before carrying out further experiments.

**Figure 3:**
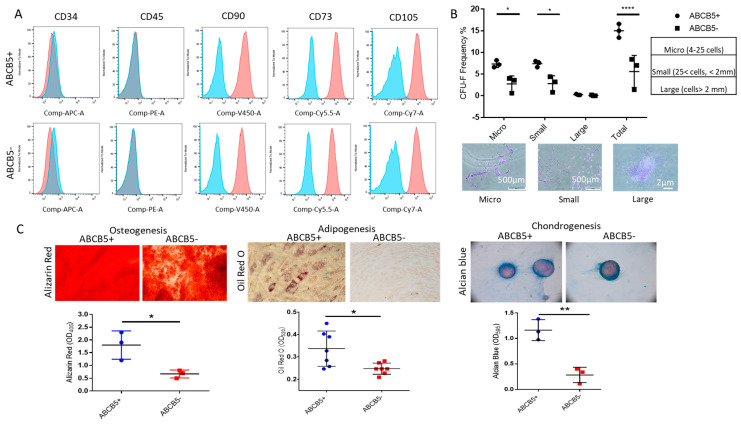
Comparison of MSC properties of ABCB5+ and ABCB5−cells. (**A**) Representative histograms of FACS analysis for a mesenchymal stem cell (positive and negative) marker panel including CD34, CD45, CD90, CD73, and CD105, demonstrating similar expression profiles for both ABCB5+ and ABCB5− cells. Blue: IgG control, Red: stained samples (**B**) Colony-forming unit efficiency comparing the percentage of micro-, small, large, and total colonies generated by ABCB5+ or ABCB5- cells and representative colony photos. (**C**) Trilineage differentiation assays using Alizarin Red (Osteogenesis), Oil Red O (Adipogenesis), and Alcian Blue (Chondrogenesis), and respective quantification of extracted dye solutions by absorbance measurements (n ≥ 3). Images captured using 20x magnification. In all graphs: * signifies *p* < 0.05, ** signifies *p* < 0.01 and **** signifies *p* < 0.0001.

First, ABCB5-positive and -negative cells were examined for MSC markers. Both cell groups did not express either CD34 or CD45, whereas they were positive for CD90, CD73, and CD105 (Figure 3A). In a colony-formation unit assay (Figure 3B), ABCB5+ cells exhibited significantly higher capacity to form micro-colonies (4–25 cells, 7.33 ± 0.81% for ABCB5+ vs. 2.73 ± 1.85% for ABCB5-, *p* < 0.05) and small colonies (cells > 25 and colony diameter < 2 mm, 7.4 ± 0.72% for ABCB5+ vs. 2.8 ± 1.83% for ABCB5-, *p* < 0.05). The numbers of large colonies were similar (0.27 ± 0.11% for ABCB5+ vs. 0.07 ± 0.11% for ABCB5-, *p* > 0.05). The total number of colonies formed by ABCB5+ cells was also significantly higher compared to their negative counterparts (15.00 ± 1.6 for ABCB5+ vs. 5.6 ± 3.704 for ABCB5-, *p* < 0.0001).

Moreover, following induction of trilineage differentiation, both cell populations were successfully differentiated to the osteogenic, adipogenic, and chondrogenic lineages, as demonstrated by positivity for Alizarin Red, Oil Red O, and Alcian Blue (Figure 3C). However, ABCB5+ cells featured significantly higher levels of all three dyes (Alizarin Red: 1.8 ± 0.56OD405 for ABCB5+ vs. 0.67 ± 0.15 OD405 for ABCB5- and *p* < 0.05, Oil Red O: 0.34 ± 0.078OD520 for ABCB5+ vs. 0.24 ± 0.024OD520 for ABCB5-, and *p* < 0.05 Alcian Blue: 1.16 ± 0.205OD595 for ABCB5+ vs. 0.28 ± 0.15OD595 for ABCB5- and *p* < 0.01, Figure 3C).

### 2.3. ABCB5+ Cells Exhibit Higher Migratory Capability in the Presence of SDF1

**Figure 4:**
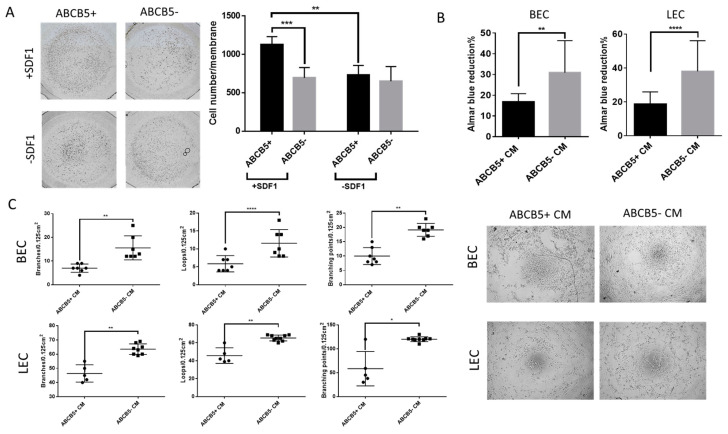
ABCB5+ cells exhibit increased migratory activity while they hamper proliferation and tube formation of blood and lymphatic endothelial cells. (**A**) Representative photographs of migrated ABCB5+ and ABCB5- cells in the presence or absence of SDF1 and respective graph depicting quantification of the migrated cell numbers (n = 5). Images captured using 5x magnification. (**B**) Alamar Blue (metabolic activity) assay of blood and lymphatic endothelial cells (BEC and LEC) treated with conditioned media from ABCB5+ and ABCB5- cells (n = 7). (**C**) Tube-formation assay representative photos and morphometric analysis by evaluation of branches, loops, and branching points of BECs and LECs treated with conditioned media from ABCB5+ and ABCB5- cells (ABCB5+ CM and ABCB5- CM, n = 3). Images captured using 20x magnification. In all graphs: * signifies *p* < 0.05, ** signifies *p* < 0.01, *** signifies *p* < 0.001 and **** signifies *p* < 0.0001.

The migratory ability of ABCB5+ and ABCB5- limbal stromal cells was examined using a Boyden chamber transmigration assay (Figure 4A). In the presence of the chemotactic protein SDF1, a significantly higher number of ABCB5+ cells migrated through the chamber membrane compared to ABCB5- cells (1129.2 ± 101.06 vs. 694.29 ± 133.721 for ABCB5+ and ABCB5- cells, respectively, *p* < 001). SDF1 had a significant stimulatory effect on the migration ability of ABCB5+ cells (1129.2 ± 101.06 vs. 730.75 ± 125.133 with and without SDF1, respectively, *p* < 0.01). Notably, SDF1 did not promote the migration of ABCB5- cells (694.29 ± 133.721 vs. 650 ± 190 for ABCB5- cells with and without SDSF1, respectively), and in its absence, the ABCB5+ and ABCB5- cells migrated in similar numbers (730.75 ± 125.133 vs. 650 ± 190 for ABCB5+ and ABCB5-, respectively).

### 2.4. The Paracrine Activity of ABCB5+ Cells Reduces the Metabolic Activity of Blood and Lymphatic Endothelial Cells and Suppresses the Proliferation of Jurkat Cells

To assess the role of the ABCB5+ stromal cell population in corneal avascularity, conditioned media from ABCB5+ and ABCB5- cells were collected and used to treat blood and lymphatic endothelial cells (BECs and LECs) in a proliferation and a tube-formation assay. Specifically, the paracrine effect on LEC and BEC metabolic activity was tested by carrying out an Alamar Blue assay.

The results, depicted in Figure 4B, indicated that the percentage reduction of the Alamar Blue reagent (indicating metabolic activity) was significantly lower in both LECs and BECs, which were treated with CM from ABCB5+ cells (18.69 ± 1.8%, vs. 38.116 ± 4.65%, *p* < 0.0005 and 16.8 ± 1.02% vs. 30.9 ± 3.99%, respectively, 48 h).

Similarly, a tube-formation assay by plating blood (BEC) and lymphatic (LEC) endothelial cells on matrigel depicted in Figure 4C indicated that compared to ABCB5+ CM, treatment with ABCB5- CM resulted in increased formed branches (BEC: 7 ± 1.32 vs. 15.57 ± 1.73, *p* < 0.01 and LEC: 46.4 ± 6.1 vs. 63.5 ± 3.72, *p* < 0.01), loops (BEC: 5.87 ± 2.23 vs. 11.57 ± 3.82, *p* < 0.0001 and LEC: 45.7 ± 8.67 vs. 65.44 ± 3.28, *p* < 0.01), and branching points (BEC: 10 ± 2.94 vs. 19.14 ± 2.23, *p* < 0.01 and LEC: 58.4 ± 36.05 vs. 120.1 ± 5.061, *p* < 0.05). Taken together, the proliferation and tube-formation assay data suggest an anti-angiogenic paracrine effect of ABCB5+ limbal stromal cells in vitro.

**Figure 5:**
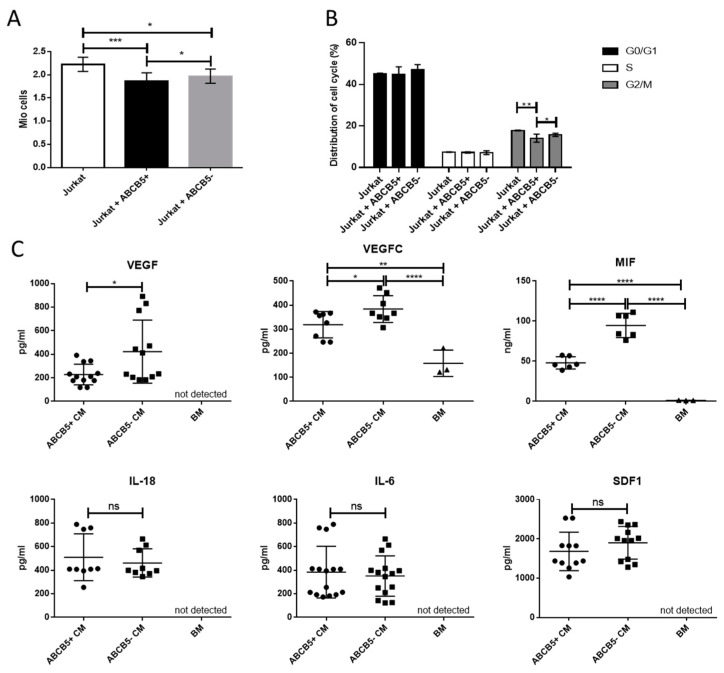
Co-culture of ABCB5+ and ABCB5- cells with Jurkat cells and ELISA of proangiogenic and proinflammatory cytokines demonstrate a comparative anti-inflammatory effect and anti-inflammatory and anti-angiogenic secreted cytokine profile of ABCB5+ cells. (**A**) Metabolic activity (Alamar Blue assay) and (**B**) cell cycle analysis of Jurkat cells co-cultured with ABCB5+ and ABCB5- cells (24 h). (**C**) Evaluation by ELISA of VEGFA, VEGFC, MIF, IL-18, IL-6, and SDF1 secreted in conditioned media of ABCB5+ and ABCB5- cells (ABCB5+ CM and ABCB5- CM, n = 5). * signifies *p* < 0.05, ** signifies *p* < 0.01, *** signifies *p* < 0.001 and **** signifies *p* < 0.0001.

In order to compare the effect of ABCB5+ and ABCB5- limbal stromal cells on T-cell proliferation and cell cycle, we co-cultured both groups of cells with Jurkat cells. After 24 h of cell culture, Jurkat cells that were co-cultured with ABCB5+ cells featured significantly lower proliferation levels compared to the control (Jurkat only) and co-cultures containing ABCB5- cells (*p* < 0.001, *p* < 0.05, proliferation rates: 1.856 ± 0.19 Mio cells, 2.27 ± 0.46 Mio cells, and 1.975 ± 0.155 Mio cells, respectively). Comparably, analysis of the distribution of cell cycle showed that co-culture with ABCB5+ cells significantly reduced the percentage of Jurkat cells in the G2/M cycle compared to the ones co-cultured with ABCB5- cells (*p* < 0.05) or the ones cultured alone (*p* < 0.01). The data are depicted in Figure 5B (14.09778 ± 1.94%, 15.689 ± 0.77%, and 17.833 ± 0.153% for Jurkat + ABCB5+, Jurkat + ABCB5-, and Jurkat only, respectively).

### 2.5. The ABCB5+ Limbal Stromal Cells Produced Lower Levels of the Pro-(Lymph)angiogenic Regulators VEGFA, VEGFC, and the Pro-Inflammatory Cytokine MIF

In order to understand the paracrine effect of the ABCB5+ stromal cells on endothelial cells and the T lymphocyte cell line, ELISA assays of key secreted angiogenesis and inflammation regulators were carried out in conditioned media (CM) from ABCB5+ and ABCB5- limbal stromal cell cultures. VEGFA and VEGFC were selected as the master regulators of angiogenesis and (lymph)angiogenesis, respectively. The selection of the pro-inflammatory cytokines MIF, Interleukin 6 (IL-6), and IL-18 was based on the results of a protein profiler human cytokine array, which was performed using CM from two donors (Supplementary Figure S1A depicting representative donor images of ABCB5+ CM, ABCB5- conditioned media, and basal media (BM) control). The cytokines with the most prominent differences after densitometry analysis of the protein array (Supplementary Figure S1B), namely SDF1, IL-6, IL-18, and MIF, were selected for further and more accurate evaluation by ELISA using multiple donors (n = 5 or higher).

VEGFA, while not detected in BM, was significantly higher in the ABCB5- CM compared to ABCB5+ CM (422.0 ± 268.4 pg/mL vs. 227.8 ± 87.84 pg/mL, *p* < 0.05). VEGFC was present in BM (158.2 ± 55.08 pg/mL), and its concentration in both ABCB5+ CM and ABCB5- CM was significantly increased (318.4 ± 54.83 pg/mL and 383.4 ± 55.8 pg/mL, *p* < 0.01 and *p* < 0.0001, respectively). The concentration in ABCB5- CM was again significantly higher compared to ABCB5+ CM (*p* < 0.05).

Moreover, while MIF was also lower in the BM (1.170 ± 0.17 ng/mL), its concentration in both ABCB5+ CM and ABCB5- CM was significantly higher (47.97 ± 7.601 ng/mL and 94.61 ± 15.04 ng/mL, *p* < 0.01 and *p* < 0.0001, respectively). The concentration in ABCB5- CM was again significantly higher compared to ABCB5+ CM (*p* < 0.05). On the other hand, there was no significant difference in the concentrations of IL-18, IL-6, and SDF1 between ABCB5+ CM and ABCB5- CM (respectively for IL-18: 509.6 ± 198.8 pg/mL and 461.6 ± 119.6 pg/mL, IL-6: 383.4 ± 219.7 pg/mL and 350.9 ± 171.1 pg/mL and SDF1: 1681 ± 488.4 pg/mL and 1900 ± 416.4 pg/mL). Neither cytokine was detected in the basal media (Figure 5C).

## 3. Discussion

The maintenance of corneal stromal homeostasis, specifically avascularity and a low-inflammatory state, is key for transparency and good vision and evolutionary highly conserved [33]. Corneal keratocytes are the main population responsible for maintaining the collagen scaffold and ECM of the corneal stroma. In the most recent years, the idea that a sub-population of these cells features stem cell characteristics has emerged, offering new avenues of understanding stromal regulation, regeneration, and cornea disease, particularly including inflammation and the scarring that leads to loss of transparency [24,34].

During development, cells originating from periocular mesenchyme constitute and maintain the corneal stroma, eventually deriving its resident keratocytes [35]. In earlier developmental stages, the corneal stroma is abundantly populated with cells that eventually undergo apoptosis, leaving only a small residual population localized in the anterior stroma, which is sufficient for the production of collagen and crystallins, which compose its translucent ECM [36]. Funderburgh et al. were the first to discover progenitor-like cells in adult corneal stromal tissue that were able to produce clones and exhibited stem cell-like characteristics [18]. These cells express Pax6 [18] and ABCG2 [37] (which are linked to their quiescent and stem cell-like properties, including multipotency) as well as the typical MSC markers CD73, CD90, CD105, and CD140b/PDGFRβ [19,20,38,39].

In this study, we identified and isolated a putative MSC population of cells derived from human corneal stroma, which express ABCB5 as well as CD73, CD90, and CD105 but are negative for CD34. Our in vitro and in situ data demonstrated the frequency of these cells in the limbal stroma. These data concur with previous reports indicating that corneal MSC cells are predominately localized in the stroma of the limbus to facilitate their crosstalk and regulation of the LESC population [26,40,41,42], which rely on this cross-talk for maintenance of LESC stemness [43,44,45,46].

This report is the first to demonstrate the expression of ABCB5 in human corneal stromal cells linked to more efficient osteogenic, adipogenic, and chondrogenic differentiation as demonstrated by quantification of Alizarin Red, Oil Red O, and Alcian Blue, respectively, as well as colony-forming unit capacity (for micro-, small, and total colonies). These data align with previous reports of an ABCB5+ MSC population found in the skin that also showed increased colony-forming and differentiation abilities in comparison to their negative counterparts [47]. Interestingly, the ABCB5+ limbal stromal cells also exhibited an increased migratory activity in a Boyden chamber assay compared to their negative counterparts. It is known that the migratory and homing capacity of MSC is key, not only in tissue homeostasis, where they migrate in case of injury or tissue renewal, but also in the context of cell therapy, where they are required to exhibit high homing efficiency when injected into tissues [48,49,50,51,52,53,54]. Intravenously administered mouse dermal ABCB5+ MSCs have emerged capable of efficiently homing to mouse skin and thymus [55]. Our data showed that the ABCB5+ limbal stromal cells only exhibited higher migratory capacity in the presence of the SDF1, a chemoattractant shown to facilitate the migration of MSC to the wound site following wound injuries in tissues, including the bone and the cornea [56,57]. In the cornea, SDF1 is produced by limbal epithelial stem cells in order to attract stromal niche cells that prevent LESC differentiation [58].

To further understand the role of the ABCB5+ cells in corneal stroma avascularity and immune privilege, we employed FACS-sorting to exclusively select ABCB5+ cells. We followed-up with functional assays where ABCB5+ cells interact with either endothelial cells or an immune cell line. It was demonstrated that CM from the ABCB5+ cells hindered proliferation and tube formation, both key morphometric characteristics of blood and lymphatic endothelial cells. Past studies have demonstrated that MSCs can conditionally facilitate or hinder angiogenesis, depending on the tissue and disease context. MSC secretome consists of a protein-soluble fraction (growth factors, cytokines) and a vesicular component (extracellular vesicles). The secretome of MSCs derived from bone marrow or adipose tissue has a promoting effect on angiogenesis [59,60,61] via factors such as VEGF, bFGF, IGF-1, and HGF [62]. MSCs used for anti-cancer treatment for solid tumors were shown to act anti-angiogenic [63]. These findings, in combination with their superior ability to home in on tumor tissues, corroborates their potential in anti-cancer cell therapy [63,64]. As previously reported, resident corneal MSCs have a paracrine anti-angiogenic effect via PEDF and sFLT-1 activity [65]. In order to understand the paracrine activity of ABCB5+ cells on endothelial and Jurkat cells, we carried out a preliminary screening by using a proteome profiler cytokine array followed-up by ELISAs for cytokines and growth factors involved in inflammatory (lymph)angiogenesis. Our data confirmed that two master regulators of angiogenesis and (lymph)angiogenesis, VEGFA and VEGFC, respectively, were significantly less abundant in ABCB5+ CM. This confirms previous reports showing that corneal MSCs produce less VEGFA, mediating a suppressing paracrine activity on endothelial cells [65]. The reduced expression of VEGFC in the ABCB5+ corneal stromal cells is a novel finding. In other MSC types such as bone marrow MSCs, for example, the expression of VEGFC actually induced (lymph)angiogenesis [66]. The reduced expression of these two factors provides an explanation for the impeding effects on BEC and LEC cells and is promising for the potential clinical relevance of ABCB5+ cells in cornea neovascularization therapy.

In addition to their reduced angiogenic effects, co-culture of ABCB5+ LESC with Jurkats, a CD4+ leukemic cell line in basal conditions (in the absence of an inflammatory stimulus), induced a mild suppressing effect on their proliferation, possibly by reducing the number of cells entering the G2/M phase. It should be noted that while this reduction of the percentage of cells in the G2/M phase is statistically significant, it is still limited possibly due to the fact that the studies are carried out in the absence of an inflammatory stimulus. Follow-up studies under inflammatory conditions will help further explore the potential suppressing effect of the ABCB5+ cells.

These data concur with previous reports demonstrating the ability of bone marrow [67,68] and adipose [69] MSCs to arrest T-cell proliferation in vitro. We compared the concentration of proinflammatory cytokines in the ABCB5+ CM and ABCB5- CM following-up the cytokine array results. It was confirmed that the concentration of MIF was lower in the CM of the ABCB5+ cells. MIF is a positive regulator of corneal (lymph)angiogenesis and is considered a pharmaceutical target for the treatment of corneal neovascularization. It is normally expressed in the corneal epithelium by stromal cells in a corneal neovascularization model, and it was shown that MIF-knockout mice featured a reduced area of neovascularization and macrophage infiltration following inflammatory challenge [70]. In a different study featuring a Pseudomonas aeruginosa-induced keratitis model, MIF induced corneal epithelial cells to produce proinflammatory cytokines. In addition, MIF directly enhances T-cell proliferation [71,72]. The fact that ABCB5+ cells secrete approximately twofold lower levels of MIF compared to their negative counterparts provides an explanation of the reduced T-cell Jurkat proliferative effect and is especially desired in the context of using these cells in cell therapy. The IFNγ inducer IL-18, usually produced by macrophages but also stressed corneal epithelial cells, also induces T-cell proliferation [73,74].

It should be noted that the ELISA data for IL-6, IL-18, and SDF1 did not confirm the differences observed in the protein array data that was used as a screening tool. This may be attributed to donor variability as well as the fact that ELISA assays allow for the simultaneous and more sensitive evaluation of multiple donor secretomes.

Due to its strategic location in the limbal stromal transition zone between avascular and immune-privileged cornea and heavily vascularized and non-immune-privileged conjunctiva, the ABCB5+ stromal MSC population may play a key role in maintaining corneal immune and angiogenic privilege [75,76]. For example, it has been reported that corneal stromal stem cells prevent neutrophil infiltration and corneal scarring by secreting TSG-6 [27]. Also, in a different study, the miRNA contained in extracellular vesicles secreted by these cells was shown to block immune cell infiltration and corneal scarring [77]. While the exact immunomodulatory mechanisms employed by these cells in resting state remains partially understood, further studies are needed to explore their role in corneal transplant immunology and maintenance of corneal avascularity.

## 4. Materials and Methods

### 4.1. Ethics Statement for the Use of Human Tissue

Human cadaveric corneal tissue, a surplus of transplantation surgery, was only used in case of priory obtaining research consent and in accordance with the declaration of Helsinki. The cadaveric tissue was obtained from the operating theatre of the Department of Ophthalmology, University of Cologne. Tissue from approximately 50 donors was used for the experiments used in this study, age ranging from 50 to 70 years and equal numbers of males and females. Due to the limited tissue availability, age and sex was distributed equally between experiments.

### 4.2. Primary Human Limbal Stromal Cell Harvesting and Maintenance

Sectioned corneal tissue was treated with a 1.2 U/mL dispase II solution (Merk, Darmstadt, Germany) for 2 h at 37 °C. Then, the epithelial cells were gently scraped by using a scalpel to be used in other projects. Subsequently, the de-epithelialized rims were dissected to pieces measuring approximately 2 mm in thickness and then placed endothelial side-up onto a 10 cm dish (Nunc). To differentiate between harvesting limbal and central corneal stromal cells, we have used either limbal tissue (as defined by approximately 1 mm from the limbal border) or central cornea tissue (biopsies measuring approximately 3 mm^2^ and excised from the center of the cornea). The explant cultures were maintained in stromal cell culture media (abbreviated as SCM, consisting of DMEM supplemented with 10% Fetal Bovine Serum (FBS) and 1% Antibiotic/Antimycotic (all from Invitrogen)), at 37 °C and 5% CO_2_ in air. Cells of fibroblastic morphology emerged 1 week following isolation and were sub-cultured by using TrypLE Express™ (all from Fischer Scientific, Schwerte, Germany) for expansion and use in experiments.

### 4.3. Maintenance of Human Lymphatic and Blood Endothelial Cells

Primary human dermal microvascular lymphatic endothelial cells (LEC; catalogue number C12217, lot number 2010909.1) and blood endothelial cells (BEC, catalogue number C12225, lot number 0100505) were purchased from PromoCell (Heidelberg, Germany) and were maintained in a supplemented ECGM MV2 culture medium according to the manufacturer’s instructions. Cells from a single donor were used in each case. The cells were passaged once, reaching 80% confluence by using a Trypsin/EDTA (0.04%/0.03%) solution for 2 min, followed by a trypsin-neutralizing solution (0.05% Trypsin Inhibitor in 0.1% BSA; both by PromoCell, Heidelberg, Germany). The cells were expanded up to passage 8. In each experiment, cells from three different passages were used.

### 4.4. FACS Sorting

Human limbal stromal cells at passage 1 were harvested with TrypLe Express (Gibco). All centrifugation steps were performed at 450 RCF for 10 min at RT. The cell pellet was re-suspended in FACS sorting buffer (consisting of Hank’s HBSS, 1 mM EDTA, 25 mM HEPES Ph = 7.1% FSC) and the cell concentration was adjusted to 5 × 10^5^ cell/250 µL. Then cells were incubated with anti-ABCB5 3C2-FITC (provided by RHEACELL, (5 µg/mL) antibody or isotype control Mouse IgG1 κ (0.5 µL) at 37 °C for 1 h, 5%. Cell suspensions were washed twice with 1 mL sorting buffer, then the pellets were re-suspended in 250 µL of FACS sorting buffer. Just before sorting, the cells were strained in Corning Falcon Test tubes with 35 µm cell strainer caps, and DAPI was added. The cell sorting was performed using a BD FACSAria Fusion at the FACS & Imaging Core Facility of the Max Planck Institute for Biology of Ageing in Cologne. Cells were collected into HLF medium. For post-sorting analysis, 100 µL of cell suspension was incubated with DAPI for 5 min then analyzed with a FACS Canto II.

ABCB5+ and ABCB5- fractions were cultured up to 60 to 90% confluence before using them for the next experiment.

### 4.5. FACS Analysis

The cells were detached by TrypLE™ Express Enzyme (1×) (Fischer Scientific, Schwerte, Germany) and counted. The cells were resuspended and adjusted to 5 × 10^5^ cell/250 µL then blocked in FACS buffer (PBS supplemented with 0.5%BSA and 2 mM EDTA) +FC Blocking reagent (Miltenyi Biotec) for 10 min at on ice, spun down, and the cell pellet was resuspended in Zombie Dye (1:10,000) (Biolegend, San Diego, CA 92121, USA) for 15 min on ice in dark. Then, the cells were incubated for 30 min at 4 °C, in the dark with the following antibodies: 10 μg/mL mouse anti-ABCB5 monoclonal antibody (Clone 3C2–1D12, as above) conjugated with FITC, CD34-APC, CD45-PE, CD90-V450, CD73-PerCP-Cy5.5, CD105-PE-Cy7 (all from Biolegend). The FMO controls were used. Finally, the cells were then washed twice with PBS supplemented with 2% FBS and were analyzed using a BD FACSCanto™ II (BD Biosciences, Heidelberg, Germany). Forward scatter (FSC) and side scatter (SSC) were used to remove cell debris and doublets, and 7AAD-negative cells were defined as live cells. The data were analyzed using the FlowJo (version 10, BD Biosciences, Heidelberg, Germany) software.

### 4.6. Mesenchymal Stem Cell Phenotypic Characterisation

#### 4.6.1. Colony-Formation Units-Fibroblast Assay

Sorted cells were seeded at a density of 500 cells/well in a six-well plate in SCM. After 10 days, the cultures were washed with PBS and fixed with 100% methanol for 5 min at RT; then, the residual methanol was washed off. Colonies were stained with 0.5% crystal violet in glacial acetic acid for 15 min. Images of the stained cells were captured by using an Olympus microscope CKX53 equipped with an Olympus EP50 camera system. The cell colonies were classified into three types: micro (4–25 cells), small (>25 cells and <2 mm), or large (>2 mm).

#### 4.6.2. Trilineage Differentiation Protocols

The cells were re-suspended in SCM, at 4000 cells/300 μL, 8000 cells/300 μL, or 8 × 10^4^ cell/5 µL for osteogenic, adipogenic, and chondrogenic differentiation, respectively, and seeded in eight chamber slides (Nunc™ Lab-Tek™ Chamber Slide System, Fischer Scientific, Schwerte, Germany). After 24 h, SCM was replaced with specialized differentiation media, either StemPro™ Osteogenesis, StemPro™ Adipogenesis, or StemPro™ Chondrogenesis (Fischer Scientific, Schwerte, Germany). The media was exchanged every 2 days with the relevant differentiation media. After 3 weeks of culture, the cells were fixed with 4% PFA for 10 min at RT.

#### 4.6.3. Oil Red O Staining

Fixed cells were incubated at RT for 15 min in Oil Red O (Oil O Red, Merk, Darmstadt, Germany) diluted in distilled water at a ratio of 6:4. Subsequently, they were rinsed four times in distilled water and imaged by using an Olympus microscope CKX53 equipped with an Olympus EP50 camera system. Quantification of the staining was done by incubating for 10 min at RT in isopropanol and on a shaker, then measuring the optical density (OD) by absorbance at 540 nm by using an Epoch spectrophotometer (BioTeK, Agilent Technologies, Santa Clara, CA 95051, USA).

#### 4.6.4. Alcian Blue Staining

For imaging: The chondro-organoids were frozen in Tissue-Tek O.C.T. compound (Sakura Finetek Europe) and cryo-sectioned. The sections were incubated for 3 min in acetic acid 3%, kept for 30 min in 0.1% Alcian Blue solution, briefly rinsed in 3% acetic acid solution, washed 10 min under tap water, rinsed in distilled water, incubated for 5 min in nuclear Fast Red solution, and finally rinsed in distilled water for 5 min prior to mounting. Images of the stained sections were taken with an Olympus microscope CKX53, equipped with an Olympus EP50 camera system.

Quantification of Alcian Blue: the fixed cultures were incubated overnight with guanidine hydrochloride (250 μL/24-well-plate well). Subsequently, the OD was measured by absorbance at 595 nm by using an Epoch spectrophotometer (BioTeK, Agilent Technologies, Santa Clara, CA 95051, USA).

#### 4.6.5. Alizarin Red Staining

Staining and concentration measurements were done by using the Alizarin Red S staining quantification assay kit (Sciencell, Carlsbad, CA 92008, USA), according to the manufacturer’s instructions. The OD was measured by absorbance at 405 nm by using an Epoch spectrophotometer (BioTeK, Agilent Technologies, Santa Clara, CA 95051, USA).

### 4.7. Immunohistochemistry

Sections were de-paraffinised, re-hydrated, and the antigens were retrieved (eBioscience™ IHC Antigen Retrieval Solution—Low pH (10×), Invitrogen). Slides were blocked for 1 h in blocking buffer (0.3% Triton, 0.4 µg/mL Human BD Fc Block™ (BD Biosciences, Heidelberg, Germany), 10% goat serum in PBS), followed by overnight incubation in (10 µg/mL) anti-ABCB5 (clone 3C2–1D12) primary antibody was applied (10 µg/mL, in blocking buffer) at 4 °C. Then, 10 µg/mL secondary antibody (Goat anti-Mouse IgG (H + L), Alexa Fluor™ Plus 555, Invitrogen) was applied for 1 h at RT in dark. Sections were washed with PBS, stained with DAPI (at a concentration of 1:2500), and mounted with DAKO mounting media.

### 4.8. Collection of Conditioned Media

The sorted cells were plated in six-well plates and at a seeding density of 10^5^ cells/well in SCM. The cultures were allowed to proliferate for 5 days, by which time they reached approximately 90% confluence. The cultures were washed once with PBS, and the cells were treated with basal media (BM) (MV2 basal endothelial medium (Promocell, Heidelberg, Germany) supplemented with 2% FBS). Conditioned media (CM) was collected after 24 h of culture, centrifuged at maximum speed to clear the dead cells and any debris, aliquoted, and stored at −80 °C for a maximum of 6 months before further use.

### 4.9. Paracrine Activity of ABCB5+ vs. ABCB5- Cells on LECs and BECs

#### 4.9.1. Alamar Blue Assay

The cells were treated with the different CM and the BM (control), as described in Section 4.8. Cell metabolic activity was evaluated by using an Alamar Blue assay (Thermo Scientific). LEC and BEC cultures were seeded at a density of 3 × 10^3^ cells per well in a 96-well plate and left overnight in complete MV2 media (Promocell, Heidelberg, Germany). Subsequently, the cells were treated with the CM for 24 h. After washing the wells with PBS, 100 μL/well Alamar Blue reagent diluted in PBS at a 1/10 ratio was added to the cells for 3 h. A minimum of five technical replicates were used. The plates were then read in an Epoch plate reader (BioTek, Agilent Technologies, Santa Clara, CA 95051, USA) in absorbance mode at 570 nm and 600 nm. The percentage of reduction of the Alamar Blue reagent was calculated according to the manufacturer’s instructions. Cell-free wells containing the Alamar Blue solution were also measured to be used as a reference/blank. These experiments were repeated with CM from a minimum of three different donors and with BECs or LECs from a minimum of two different passages.

#### 4.9.2. Tube-Formation Assay

The tube-formation assays were performed on Matrigel (Merk, Darmstadt, Germany) in μ-Slide angiogenesis assay (Ibidi) according to the manufacturer’s instructions. Then, BEC or LEC were seeded at a cell density of 1 × 10^4^ cells/well in a complete MV2 media (Promocell, Heidelberg, Germany). One hour later, the cells fully adhered on the Matrigel, and the media was replaced with either ABCB5+ CM, ABCB5- CM, or BM (control) (n = 5). The tube networks formed were photographed after 16 h using a Zeiss Primo Vert inverted microscope fitted with an AxioCam ERc5s camera (Zeiss, Oberkochen, Deutschland). The number of branches, loops, and branching points were quantified by hand in Image J freeware (available for download from https://imagej.net/ij/ (accessed on 27 August 2024)). The experiments were performed a minimum of three times with LEC and BEC cells of three different passages using supernatants from different donors.

### 4.10. Co-Culture of Limbal Stromal Cells with Jurkat Cells and Measurement of Proliferation, Cell Cycle

The Jurkat cell line (kindly offered by the laboratory of Professor Thomas Langer, Max Planck Institute for Biology of Ageing, Cologne, Germany) was maintained in RPMI 1640 medium (Gibco) with 10% FBS, 100 U/mL penicillin, and 100 mg/mL AA. The culture medium was replaced every second day. The ABCB5 sorted limbal cells and Jurkat cells were co-cultured for subsequent experiments in RPMI in a 0.4-µm 24-well Transwell system (Merk, Darmstadt, Germany), where the limbal stromal cells were seeded in the upper chamber and Jurkat cells in the lower chamber at a ratio of 1:5. The Jurkat cells were seeded at a density of 10^5^ cells/mL per well in 600 μL of medium.

For cell proliferation: at 24 h, the Jurkat cells were collected in an Eppendorf tube and washed with PBS, then incubated in 200 μL Alamar Blue reagent diluted in PBS at a ratio of 1/:0 for 3 h; The Alamar Blue solution was transferred into a plate and read in an Epoch plate reader (BioTek, Agilent Technologies, Santa Clara, CA 95051, USA) in absorbance mode at 570 nm and 600 nm. A standard curve was set up to allow for quantification of Jurkat cell numbers in the different groups.

For cell cycle: at 24 h, the Jurkat cells were collected, washed with PBS, and then fixed in 2% PFA for 30 min on ice. After washing twice in PBS, the cells were stained with DAPI for 30 min at RT. The PI was added, and the cells were analyzed by using a BD FACSCanto™ II (BD Biosciences, Heidelberg, Germany).

### 4.11. Cell Migration Assay

Sorted cells at density 5 × 103 were seeded in the upper chamber of the Transwell system (8 µm pore polycarbonate membrane insert). The lower chamber was human SDF1 (Peprotech, NJ, USA) at final concentration 100 ng/mL. After 24 h of incubation, the upper part of the membrane was gently scraped with a cotton swab to remove non-migrating cells. Then, it was washed with PBS. After that, the membrane was fixed with 3.7–4% formalin overnight at 4 °C and stained with haematoxylin for 4 h at room temperature.

### 4.12. Cytokine Asessment in ABCB5+ and ABCB5- Secretome

#### 4.12.1. Protein Array

CM of ABCB5+ and ABCB5- cells from two donors and the control BM were analyzed in a Proteome Profiler Human Cytokine Array (R&D Biosciences) according to the manufacturer’s instructions. The arrays were visualized using a ChemiDocXRS+ System, and the signal was semi-quantified by densitometry using the ImageJ software (https://imagej.net/ij/). The reference spots in each membrane were used for signal normalization.

#### 4.12.2. ELISA

Enzyme-linked immunosorbent assay kits from bio-techne (Minneapolis, MN 55413, USA) were used for protein analysis according to the manufacturer’s instructions. VEGFA, VEGFC, MIF, IL18, IL6, and SDF1 were quantified using the respective human Quantikine ELISA. Each sample was analyzed in triplicate. The proteins were quantified in CM from at least five donors.

### 4.13. Statistical Analysis

Statistical analysis of the experimental data was carried out by using Prism 6.0 software (GraphPad, Boston, MA 02110, USA). Depending on the case a one-way ANOVA or a *t*-test with Mann–Whitney post-test was applied. Results producing a *p* value lower than 0.05 were defined as statistically significant. We used a minimum of three experimental triplicates. Cells from at least three cadaveric tissue donors were used to account for biological variability. Error bars displayed in graphs correspond to standard deviation. Stars displayed in graphs correspond to statistical significance as follows: * for *p* < 0.05, ** for *p* < 0.01, *** for *p* < 0.001, and **** for *p* < 0.0001.

## 5. Conclusions

This study describes a limbal stromal mesenchymal stem cell population expressing ABCB5. These mesenchymal stem cells have reduced expression of key pro-(lymph)angiogenic and proinflammatory factors and displayed both significantly reduced pro-(lymph)angiogenic and pro-inflammatory paracrine activity compared to their negative counterparts. This demonstrates that they are a unique corneal stromal sub-population and makes them an attractive alternative for anti-angiogenic/anti-inflammatory cell therapy in the cornea, which merits further pre-clinical investigation.

## Data Availability

All data are included in the article and Supplementary Materials. Further information or data can be made available upon request.

## Supplementary Materials

The following supporting information can be downloaded at: https://www.mdpi.com/article/10.3390/ijms25179702/s1.
