# Supplementary material for: Anti-Inflammatory and Anti-(Lymph)angiogenic Properties of an ABCB5+ Limbal Mesenchymal Stem Cell Population
Source: Int J Mol Sci. 2024 Sep 7;25(17):9702. doi: 10.3390/ijms25179702 (PMC11395824; doi:10.3390/ijms25179702)
Supplement: Supplementary file 1 [file ijms-25-09702-s001.zip › ijms-3156217-supplementary.pdf]

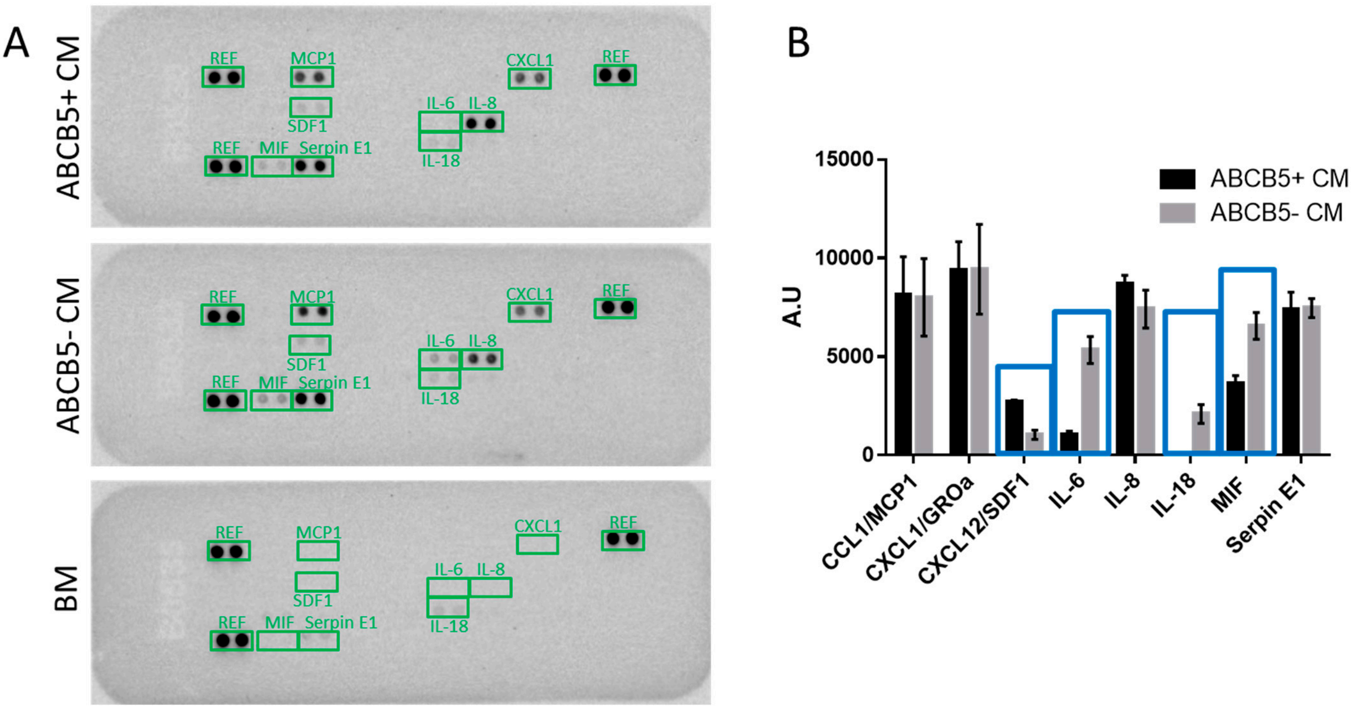

Supplementary Figure 1. Proteome Profiler cytokine array comparing the secretome of ABCB5+ and ABCB5-. *A) Representative cytokine array membranes for ABCB5+ CM, ABCB5- CM and BM and B) Array signal quantification for individual target proteins using densitometry. The blue squares highlight the cytokines with the most pronounced differences between the two groups. Statistical significance was not calculated in this screening test as CM from only two donors was used.*
